# Epstein–Barr virus oncoprotein–driven B cell metabolism remodeling

**DOI:** 10.1371/journal.ppat.1010254

**Published:** 2022-02-02

**Authors:** Eric M. Burton, Benjamin E. Gewurz

**Affiliations:** 1 Division of Infectious Diseases, Department of Medicine, Brigham and Women’s Hospital, Boston, Massachusetts, United States of America; 2 Harvard Program in Virology, Harvard Medical School, Boston, Massachusetts, United States of America; 3 Broad Institute of Harvard and MIT, Cambridge, Massachusetts, United States of America; 4 Department of Microbiology, Harvard Medical School, Boston, Massachusetts, United States of America; University of Arizona, UNITED STATES

The γ-herpesvirus Epstein–Barr virus (EBV) persistently infects >95% of adults worldwide and contributes to 200,000 cancers annually [1]. EBV uses latency programs to convert metabolically quiescent B lymphocytes into blasts that enter germinal centers and differentiate into memory B cells, the reservoir for lifelong infection. Over the first 3 days of infection, EBV expresses the pre-proliferation program, where particularly high levels of Epstein–Barr nuclear antigen 2 (EBNA2) and its key host target MYC induce metabolism pathways needed for B cell remodeling and proliferation (**Fig 1**) [1–4]. Over this period, infected cells quadruple in volume in preparation for hyperproliferation [2,5], reminiscent of remodeling that fuels germinal center centroblasts [6]. The EBV latency IIb program, comprised of 6 EBNAs and noncoding RNA (ncRNA), then supports hyperproliferation between days 4 and 7 postinfection in cell culture (**Fig 1**). Subsequently, cells convert to lymphoblastoid physiology, where 6 EBNAs and 2 latent membrane proteins (LMPs) further remodel host metabolism [7,8]. If left unchecked by immune surveillance, latency III causes outgrowth of lymphoblastoid cell lines (LCLs) that model posttransplant lymphoproliferative diseases (PTLDs) and central nervous system lymphomas [1] (**Fig 1**). Here, we review key host metabolism pathways subverted by EBV oncoproteins, with a focus on B cell transformation.

**Fig 1:**
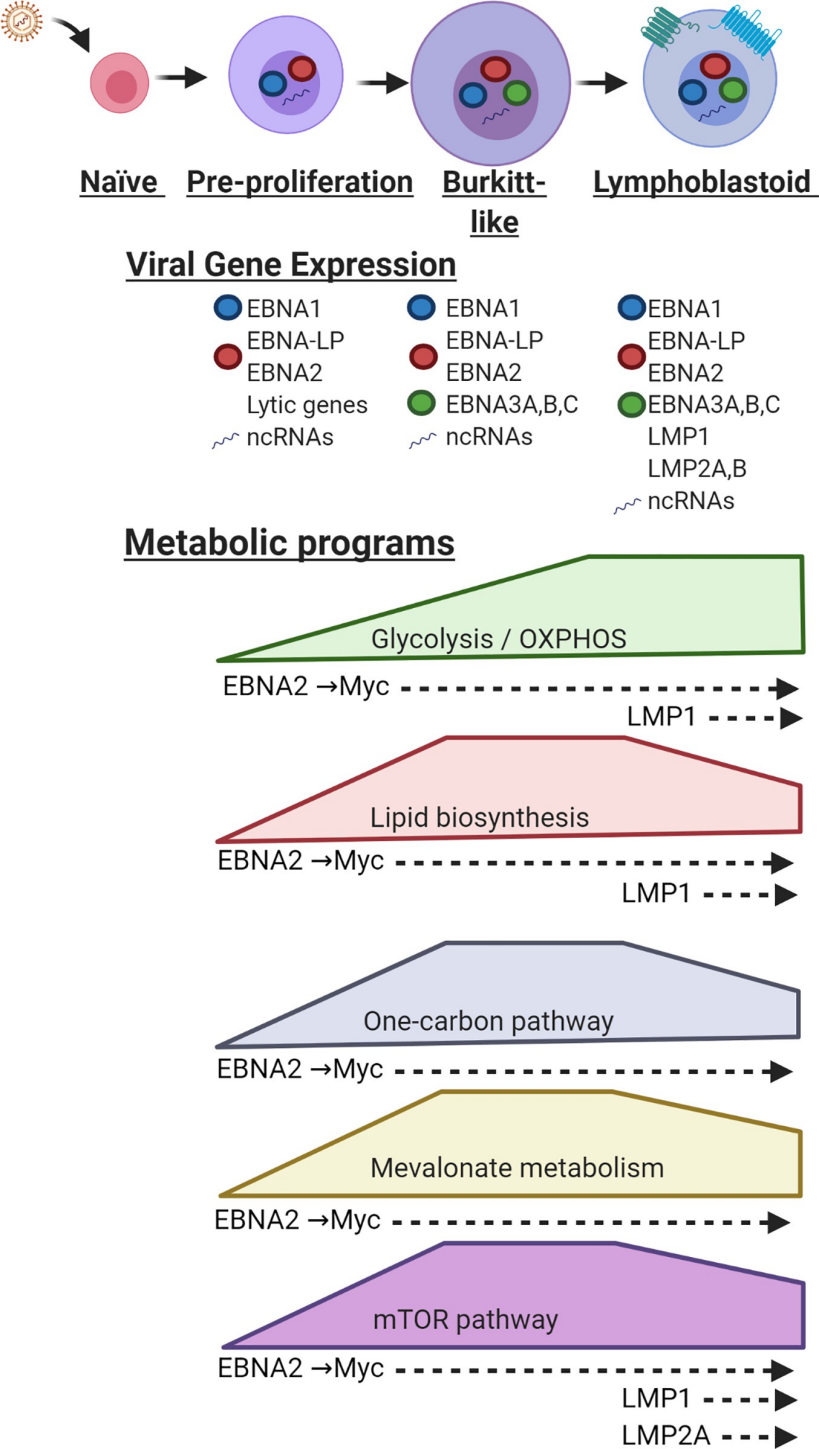
EBV latency oncoprotein–driven B cell metabolism remodeling. Schematic diagram demonstrating changes in key metabolic pathways during EBV-driven B cell transformation into LCLs. Relative cell size and pathway activation states are depicted. EBV factors associated with each phase of transformation are noted. Metabolic programs and EBV oncogenic factors that regulate them are displayed below. EBNA, Epstein–Barr nuclear antigen; EBV, Epstein–Barr virus; LCL, lymphoblastoid cell line; LMP, latent membrane protein; ncRNA, noncoding RNA; OXPHOS, oxidative phosphorylation.

## Latent EBV induction of aerobic glycolysis

Within 4 days of infection, EBV highly induces glucose uptake and expression of all glycolysis enzymes at the mRNA and protein levels, in particular the first and rate-limiting enzyme hexokinase 2 [2,4,9]. Seahorse assays confirm that EBV increases glycolytic flux [4,9], which generates lower quantities of ATP than oxidative phosphorylation (OXPHOS), but provides key building blocks for anabolic metabolism. EBNA2, the only EBV oncoprotein required for newly infected B cell outgrowth over the first 8 days of in vitro infection [10], is required for glycolytic enzyme induction, likely together with MYC [4,10].

Prior to B cell proliferation, EBV down-regulates thioredoxin-interacting protein (TXNIP), a key negative regulator of the glucose transporter GLUT1 [4,9]. TXNIP is also down-regulated upon lytic reactivation [4,11,12]. GLUT1 plasma membrane abundance is further supported by LMP1, which mimics CD40 to activate NF-κB, MAPK, PI3K/AKT, and interferon regulatory factors [13–16]. Of these, canonical NF-κB and AKT are necessary for GLUT1 up-regulation [13–16]. LMP1 also promotes glycolysis through HIF1α, which it induces through MAPK signaling and by generation of reactive oxygen species [17–19]. LMP1-activated Poly(ADP-ribose) polymerase 1 (PARP1) coactivates HIF1α-dependent gene expression, including via the formation of a complex with PARylated HIF1α at target promoter regions. LMP1-driven aerobic glycolysis is also observed in nasopharyngeal carcinoma tumor cells in culture [17–21]. Similarly, in latency III B cells, EBNA-LP and EBNA-3A further prevent HIF1α hydroxylation and degradation via association with prolyl hydroxylases 1 and 2, respectively [22].

Conversion of glucose to pyruvate produces NADH. To maintain redox balance and avoid reductive stress, NAD must be regenerated in cells with high glycolysis flux. This can occur by conversion of pyruvate to lactate or by OXPHOS, each of which oxidizes NADH to NAD, and each of which are important for EBV-mediated B cell transformation. To facilitate lactate release, EBNA2 and LMP1 induce expression of the monocarboxylate transporters MCT1 and MCT4, respectively [23].

## EBNA2 and LMP1 induce oxidative phosphorylation

EBV strongly induces OXPHOS upon B cell infection [2,4,9], likely needed in addition to glycolysis to meet ATP demand. EBV-induced OXPHOS also likely serves to maintain cellular NAD/NADH redox balance, to support de novo pyrimidine synthesis at the level of the flavin-dependent enzyme dihydroorotate dehydrogenase (DHODH) and to regenerate ubiquinone/coenzyme Q for redox defense. OXPHOS genes are encoded by the host nuclear genome and therefore accessible to EBNAs and to LMP-activated host transcription factors. OXPHOS remodeling coincides with EBV-driven increases in oxygen consumption rates [4,9]. Underscoring the importance of EBV-driven OXPHOS, chemical inhibition of mitochondrial respiration impairs proliferation of newly EBV-infected primary human B cells [4,9]. Likewise, switching the media sugar source from glucose to galactose, which is metabolized by OXPHOS rather than by glycolysis, slows but does not prevent EBV-driven B cell outgrowth [4,9]. Furthermore, metabolic stress is a barrier to the outgrowth of EBV-infected B cells in vitro, where a population of EBV-infected cells growth arrest exhibit reduced OXPHOS and TCA activity. Anaplerotic supplementation of TCA intermediates restores proliferation in a subset of these arrested cells [9]. Autophagy also serves to manage metabolic stress in EBV-transformed cells [9,15,24].

## EBV-driven purine and pyrimidine nucleotide metabolism

**Fig 2:**
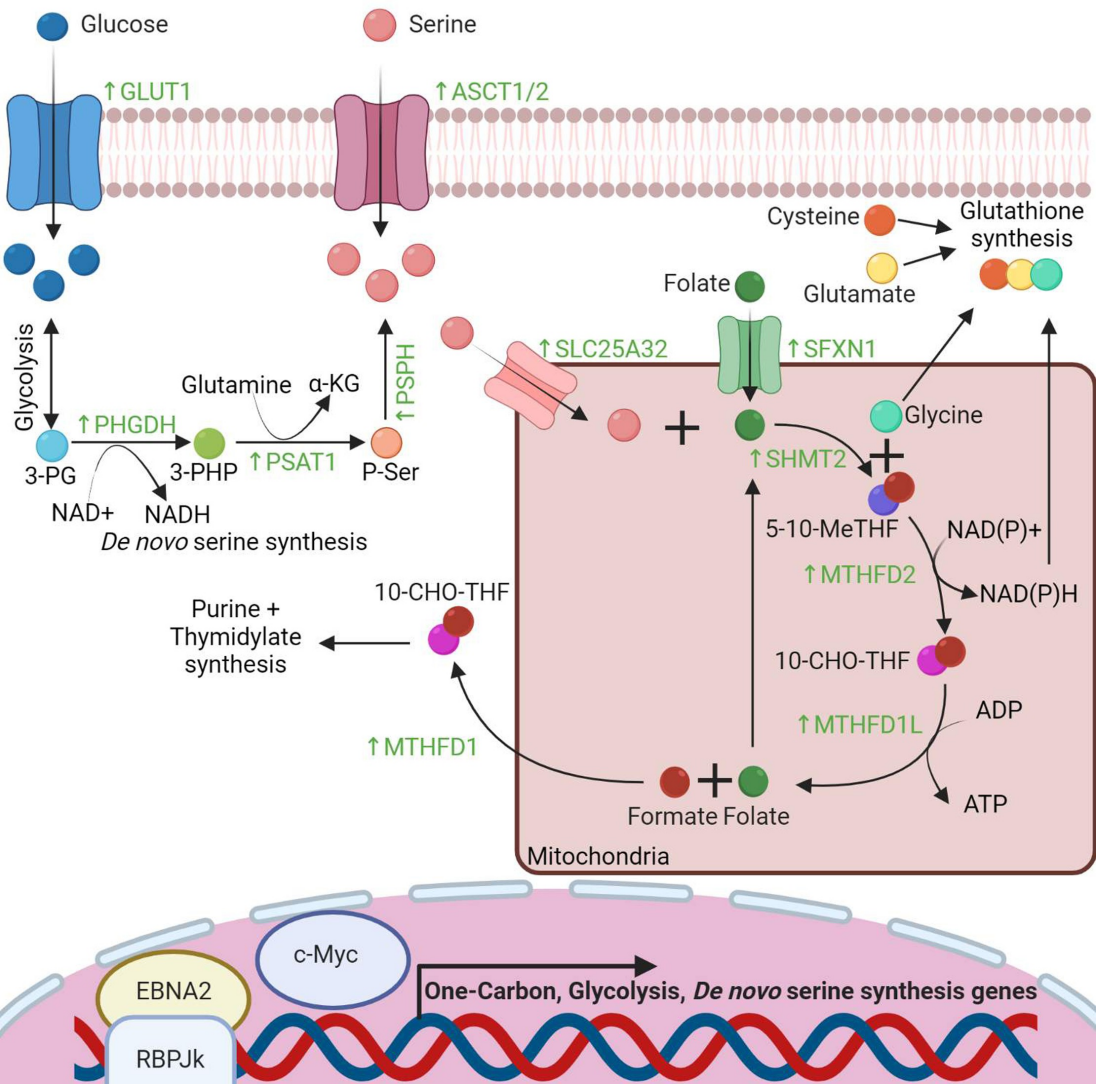
EBV-driven 1C pathway drives nucleotide and glutathione synthesis. EBNA2 and MYC induce the 1C, glycolysis, and de novo serine synthesis pathways in B cells. They also up-regulate plasma membrane abundance of the GLUT1 and ASCT1/2 transporters to increase glucose and serine import, respectively. Serine pools are further expanded by the de novo serine synthesis pathway, which metabolizes the glycolysis product 3-PG into serine and generate NADH and αKG byproducts. EBV also supports mitochondrial 1C metabolism via up-regulation of the SLC25A32 serine and SFXN1 folate transporters. Mitochondrial 1C metabolism converts serine into glycine, NADPH, and a serine-derived carbon unit (red ball), which is shuttled into the cytosol for use in purine and thymidylate biosynthesis. The carbon-loaded 1C folate carriers 5-10-meTHF and 10-CHO-THF are shown. 1C, one-carbon; 3-PG, 3-phosphoglycerate; 3-PHP, 3-phosphohydroxypyruvate; P-ser, 3-phosphoserine; 5-10-meTHF, 5-10-methylenetetrahydrofolate; 10-CHO-THF, 10-formyl THF; EBNA2, Epstein–Barr nuclear antigen 2; EBV, Epstein–Barr virus.

Nucleotide synthesis rates are low in resting B lymphocytes, which reside in a Go cell cycle state. Therefore, EBV must rapidly induce purine and pyrimidine biosynthesis for genome replication as well as ribosomal RNAs to support elevated translation rates in newly infected cells [2]. To meet purine and thymidylate demand, EBV induces the mitochondrial one-carbon (1C) pathway, which metabolizes serine into glycine and a carbon unit that can be used for downstream biochemical reactions [4] (**Fig 2**). Tracing studies demonstrate that 1C serine metabolism strongly contributes to purine and thymidylate pools in Burkitt-like hyperproliferation. Mechanistically, EBNA2 and MYC bind to the promoter of the host gene *MTHFD2* and up-regulate its expression to increase flux through the mitochondrial 1C pathway [4]. Nonetheless, limitations in purine pools induce replication stress and DNA damage, which can be reduced by nucleotide supplementation [9,25,26]. EBV also progressively induces the purine metabolism enzyme adenosine deaminase (ADA) in newly infected B cells in vitro [27]. Notably, ADA catalyzes the hydrolysis of adenosine to inosine and plays key roles in adenosine metabolism in germinal centers, where it serves to prevent the accumulation of deoxyadenosine triphosphate to toxic levels [28]. ADA levels progressively increase in EBV-infected cells, particularly as they convert to the lymphoblastoid phase [27]. Thus, EBV-induced ADA may serve to protect infected B cells as they transition through germinal center reactions and again in lymphoblastoid B cells, where ADA knockdown impairs proliferation in vitro [27]. Mechanistically, EBNA1 binds to an enhancer upstream of the *ADA* gene and is sufficient for ADA up-regulation in TERT-immortalized nasopharyngeal cells, but not in EBV-negative B cell lines, perhaps because of their higher basal ADA levels.

The pyrimidine cytidine nucleotide triphosphate (CTP) is critical for DNA, RNA, and phospholipid synthesis as well as for protein sialyation. To meet demand, EBNA2, MYC, and the LMP1-activated noncanonical NF-κB pathway highly up-regulate the rate-limiting enzyme cytidine 5′ triphosphate synthase 1 (CTPS1). EBV also up-regulates the isozyme CTPS2, which plays a partially redundant role with CTPS1 in EBV-transformed B cells. Interestingly, Burkitt and lymphoblastoid cells can utilize cytidine salvage metabolism, in which imported uridine or cytidine are converted to CTP [29]. This plasticity may underlie the clinical observation that EBV+ lymphomas frequently occur in patients with inborn hypomorphic CTPS1 mutations that severely impair T/NK cell immunity [30]. By contrast, inhibition of the enzyme DHODH, which supports de novo thymidylate and CTP biosynthesis, restrains EBV-transformed B cell proliferation and lytic replication in vitro and outgrowth of EBV+ lymphomas in humanized mice [31].

## Latent EBV induction of amino acid metabolism

Plasma membrane proteomic analysis identified amino acid transporters as among the most highly EBV-induced host proteins upon primary human B cell infection [4]. These include the ASCT1/2 and LAT1 neutral amino acid transporters (**Fig 2**) and the xCT (also called SLC7A11) cystine/glutamate antiporter, which reach near maximal levels by day 4 post-EBV infection at the RNA and protein levels [2,4,32]. Transporter induction supports rapid amino acid influx, which likely serves to activate mTOR, to participate in redox defense, and to support myriad anabolic reactions [33,34]. At later time points, LMP1 and LMP2A further activate the PI3K/mTOR pathway, and LMP1 also promotes glutamine uptake in nasopharyngeal carcinoma (NPC) cells [35]. αKG is also produced by EBV-induced de novo serine synthesis (**Fig 2**) and has key roles in metabolic reactions, including in the TCA cycle and in EBV-induced TET2 DNA demethylase activity critical for latency III [35–37].

## Latent EBV subversion of host lipid metabolism pathways

The mevalonate and fatty acid biosynthesis pathways are highly EBNA2 and MYC induced in newly infected primary human B cells [4,5]. These pathways use glucose-derived acetyl-CoA, together with NADPH reducing power, to produce sterols, isoprenoids, cholesterol, and fatty acids. The mevalonate pathway product geranylgeranyl pyrophosphate (GGPP) then plays a key cross talk role in support of LMP1 and LMP2A. Following EBNA3A/C-mediated up-regulation of the GTPase Rab13 expression [5,38], covalent GGPP modification licenses Rab13 to chaperone LMP1 and LMP2A to membrane signaling regions, thereby providing a metabolic link between multiple EBV latency III oncoproteins [5].

EBV also up-regulates the rate-limiting fatty acid synthesis enzymes acetyl-CoA carboxylase 1 (ACACA) and fatty acid synthase (FASN), which convert acetyl-CoA into palmitate for palmitoylation, triglyceride, and long-chain fatty acid pathways [5]. EBNA2, MYC, and sterol response element binding protein 2 (SREBP2) are each critical for ACACA and FASN induction. LMP1 further supports FASN up-regulation [39]. Similarly, LMP1 induces sterol response element binding protein 1 (SREBP1) in NPCs to up-regulate FASN and promote tumor progression, and LMP1 and FASN levels correlate in primary NPC samples [40]. Since CRISPR analysis indicates that SREBP2, rather than SREBP1, is an LCL dependency factor, EBV latency program and/or cell type may dictate which SREBP drives EBV-infected lipid metabolism. Upon lytic reactivation, the EBV immediate early gene RTA also up-regulates FASN, and FASN inhibition disrupts EBV lytic replication [41].

High rates of lipid biosynthesis raise the question of how EBV-infected cells are protected against ferroptosis, a programmed cell death pathway driven by iron-catalyzed lipid metabolism–generated reactive oxygen species. EBV-mediated metabolism reprograming likely supports redox defense, given that 1C supplies NADPH and glycine building blocks for antioxidant glutathione synthesis. Indeed, metabolic tracing studies demonstrate important 1C roles in support of glutathione biosynthesis and for the 1C enzyme MTHFD2 in supporting NADPH levels in newly infected B cells [4] (**Fig 2**).

## Concluding remarks

Despite recent advances, key questions remain to be addressed about how EBV remodels host cell metabolism to support infected cell growth and survival, including in human cancers with characteristic latency programs. How EBV-driven metabolism pathways affects the microenvironment, and how this, in turn, might alter antiviral T and NK cell responses, is largely unstudied. It will also be of interest to define how EBV specifically alters infected B cell immunometabolism within germinal centers to cope with relative hypoxia and nutrient constraints in this specialized lymphoid microenvironment. We speculate that as infected cells transit through germinal centers and switch to the viral latency II program, comprised of EBNA1, LMP1, LMP2A, and ncRNAs (**Fig 1**), they increase their reliance on glycolysis and lactate secretion at the expense of OXPHOS to cope with hypoxia. How EBV latency II more broadly remodels B cell metabolism remains an open question, which has been limited by a paucity of latency II model systems. Newly developed tonsil organoid and humanized mouse models promise new insights into the metabolism of B cells in this germinal center latency II state [42]. With regard to latency I, little is presently known about whether EBV alters host cell metabolism in memory B cells to support this reservoir for lifelong infection. Although EBV+ memory B cell models are needed, single-cell analyses of human tonsil samples may offer new insights into metabolism pathway states in EBV+ versus uninfected memory cells. With respect to EBV strains, little is presently known about how type I versus II EBV differentially alter infected B cell metabolism and how this contributes to observed differences in growth transformation and lytic gene expression. A key area will be to specifically define how EBNA2 polymorphisms, including one that contributes to superior type I growth transforming activity [43], affect flux through host metabolism pathways described above.

Enhanced understanding of EBV-driven metabolism network remodeling promises to lay the groundwork for new therapeutic approaches to EBV+ lymphomas. For instance, HMG-CoA reductase inhibitors (statins) block EBV-induced mevalonate metabolism and limit outgrowth of EBV-transformed B cells in culture [2,5], suggesting that it would be of interest to test these widely used medications in murine PTLD models. Similarly, given robust EBV-driven 1C pathway up-regulation, a next step will be to test 1C antagonists in xenograft and PTLD models in vivo. CRISPR analysis and small molecule studies suggest that the major metabolism regulator PI3K/AKT pathway is critical for lymphoblastoid B cell survival [44,45]. CRISPR screens also identified that Burkitt cells rely on the lysosomal iron reductase CYB561A3 to reduce transferrin-imported iron to bioavailable ferrous (Fe^2+^) iron [46]. While effects on nontransformed cells remain to be defined, nearly 700 Cancer Dependency Map cell lines derived from a wide range of human tissues were not dependent on CYB561A3 for proliferation, suggesting that a therapeutic window may exist for CYB561A3 antagonists in Burkitt lymphoma treatment. LCLs instead use STEAP3 to reduce imported lysosomal iron [46], indicating that STEAP3 may be a druggable PTLD target. How EBV-driven metabolism remodeling controls the maintenance of restricted forms of EBV latency at the level of cross talk with DNA and histone methylation remains incompletely understood. It is plausible that metabolism pathways can be exploited to derepress highly immunogenic latency III or lytic cycle antigens in order to sensitize EBV+ tumor cells to immunotherapy or lytic induction approaches, respectively. Finally, latent EBV infection can sensitize cells to synthetic lethal combinations that target multiple EBV oncometabolism pathways. For instance, blockade of EBV-induced MCT lactate exporters and electron transport chain complex I is highly toxic to LCLs [23].
