# Correction: Epstein–Barr virus oncoprotein–driven B cell metabolism remodeling

**DOI:** 10.1371/journal.ppat.1010920

**Published:** 2022-10-20

**Authors:** Eric M. Burton, Benjamin E. Gewurz

Fig 2 is incorrect. The labels for SLC25A32 and SFXN1 are swapped. The authors have provided a corrected version of Fig 2 here.

**Fig 2:**
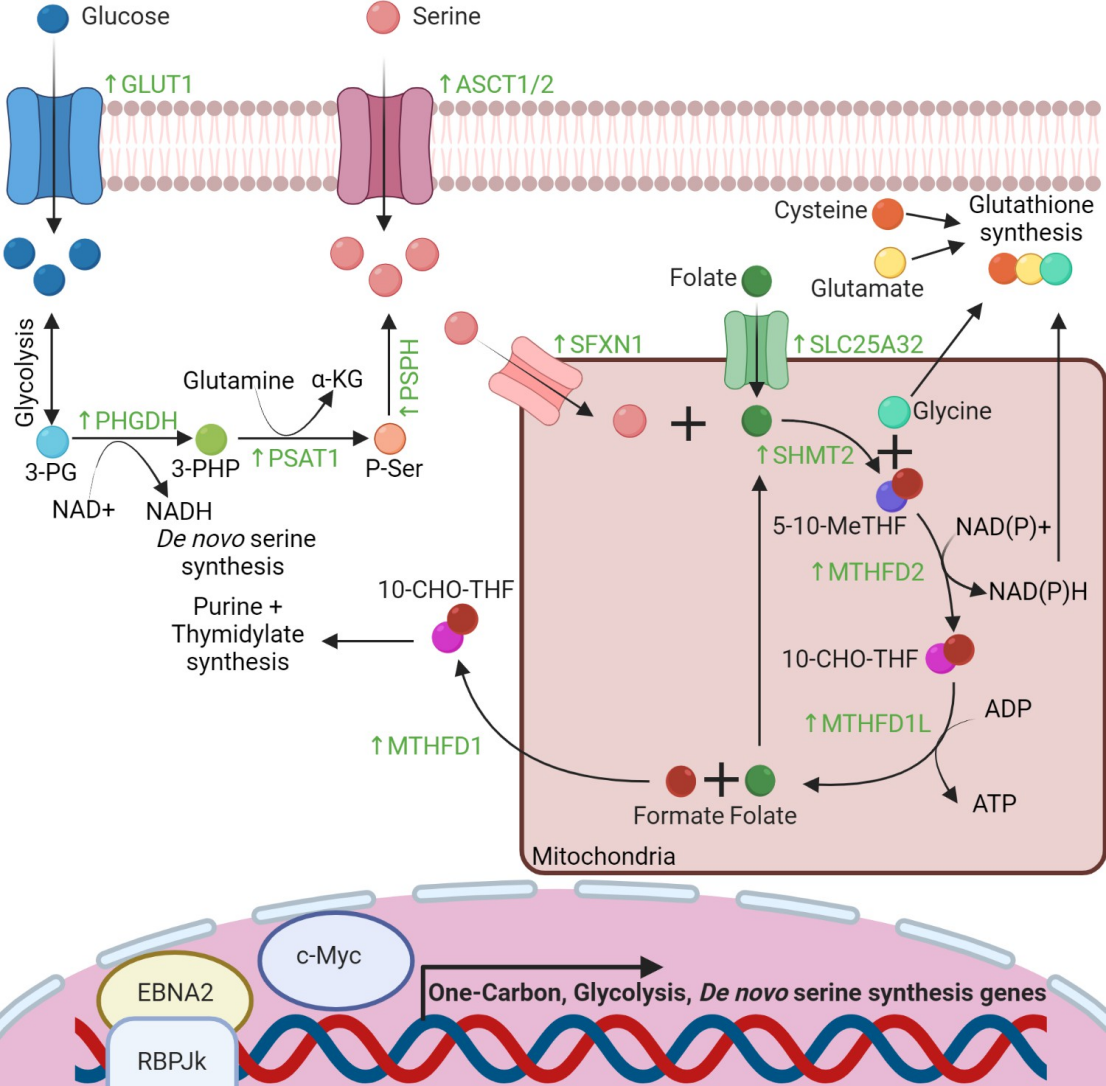
EBV-driven 1C pathway drives nucleotide and glutathione synthesis. EBNA2 and MYC induce the 1C, glycolysis, and de novo serine synthesis pathways in B cells. They also up-regulate plasma membrane abundance of the GLUT1 and ASCT1/2 transporters to increase glucose and serine import, respectively. Serine pools are further expanded by the de novo serine synthesis pathway, which metabolizes the glycolysis product 3-PG into serine and generate NADH and αKG byproducts. EBV also supports mitochondrial 1C metabolism via up-regulation of the SLC25A32 serine and SFXN1 folate transporters. Mitochondrial 1C metabolism converts serine into glycine, NADPH, and a serine-derived carbon unit (red ball), which is shuttled into the cytosol for use in purine and thymidylate biosynthesis. The carbon-loaded 1C folate carriers 5-10-meTHF and 10-CHO-THF are shown. 1C, one-carbon; 3-PG, 3-phosphoglycerate; 3-PHP, 3-phosphohydroxypyruvate; P-ser, 3-phosphoserine; 5-10-meTHF, 5-10-methylenetetrahydrofolate; 10-CHO-THF, 10-formyl THF; EBNA2, Epstein–Barr nuclear antigen 2; EBV, Epstein–Barr virus.
